# Supplementary material for: What Genetics Can Do for Oncological Imaging: A Systematic Review of the Genetic Validation Data Used in Radiomics Studies
Source: Int J Mol Sci. 2022 Jun 10;23(12):6504. doi: 10.3390/ijms23126504 (PMC9224495; doi:10.3390/ijms23126504)
Supplement: Supplementary file 1 [file ijms-23-06504-s001.zip › ijms-1740797-supplementary.pdf]

Table S1. Radiogenomics in practice - What can genetic sciences do for clinical imaging in patients with Gliomas/Astrocytomas

[Ref.]	Utility/ Outcome Measured	Mutations/ Genes	Molecular /Biological Processes	Main Results or Finding
[1]	Prognosis / OS	1740 genes classified GBM into subtypes: pro-neural, neural, classical, and mesenchymal	Homeostasis and cell cycling pathways.	Volumetric features were significantly associated with diverse sets of biological processes (false-discovery-rate < 0.05). While necrosis (NE) and tumor bulk (TB) were enriched for immune response pathways and apoptosis, contrast enhancement (CE) was associated with signal transduction and protein folding processes. Edema (ED) was mainly enriched for homeostasis and cell cycling pathways. ED was also the strongest predictor of molecular GBM subtypes (AUC = 0.61). CE was the strongest predictor of overall survival (C-index = 0.6; Noether test, p = 4x10−4).
[2]	Prognosis / OS	IDH mutation	Regulation of NFκB transcription-factor activity, dendrite morphogenesis, apoptosis, macrophage activation, immune cell activity	Each voxel is either high or low intensity on MRI sequences T1, T1c, T2, and Flair creating 16 habitats (2 to the power of 4). Imaging habitats 2, 7, and 10 were significant (p < 0.05) for determining OS after adjustment. Habitat 2 was associated with necrosis (p = 0.0172). Habitat 2 was positively associated with positive regulation of NFκB transcription-factor activity, while negatively associated with dendrite morphogenesis. Habitat 7 correlates positively with DNA damage response signal transduction, resulting in apoptosis and macrophage activation induction. Habitat 7 was correlated negatively with immune cell activity (monocyte differentiation). Habitat 10 had a positive association with signal transducers and activators of transcription-1 (STAT-1) and Natural killer cell activation while negatively correlated with ion channel activity (potassium channel inhibitor activity and voltage-gated calcium channel activity).
[3]	Prognosis / OS	Metagenes: WDR72, C14orf39, TIMP1, CHIT1, ROS1 EREG		The GBDT model with 72 features with the highest importance had the highest accuracy of 0.81 on both short and long survival time classes, and the AUC for short and long survival time classes was 0.79 and 0.81. Six metagenes showed significant interactive effect (P < 0.05), and Pearson's correlation analysis revealed that three of these metagenes (TIMP1, ROS1, EREG) showed moderate (0.3 < ∣r∣ < 0.5) or high correlation (∣r∣ > 0.5) with image features.
[4]	Prognosis / OS	MGMT promoter methylation		The fusion radiomics signature exhibited supreme power for predicting MGMT promoter methylation, with an AUC of 0.925 in the training cohort and 0.902 in the validation cohort. The performance of the radiomics signature surpassed that of clinical factors and ADC parameters. Moreover, the radiomics approach successfully divided patients into high-risk and low-risk groups for overall survival after TMZ chemotherapy for patients with Astrocytoma (p = 0.03).
[5]	Prognosis / OS	IDH mutation, 1p/19q codeletion, ATRX mutation	Hypoxia, angiogenesis, apoptosis, and cell proliferation	The six-feature radiomic signature stratified patients in the training cohort into low- or high-risk groups of overall survival (p = 0.0018). This result was successfully verified in the validation cohort (p = 0.0396). Radiogenomic analysis revealed that the prognostic radiomic signature was associated with hypoxia, angiogenesis, apoptosis, and cell proliferation. The nomogram resulted in high prognostic accuracy (Training cohort C-index: 0.92, Validation cohort C-index: 0.70) and favorable calibration for individualized survival prediction in the training and validation cohorts.
[6]	Prognosis / OS	Hypoxia-associated genes		Using the expression profile of 21 hypoxia-associated genes, a hypoxia enrichment score (HES) was obtained for the training cohort of 85 cases. Mutual information score was used to identify a subset of

				radiomic features that were most informative of HES within 3-fold cross-validation to categorize studies as short-term survival (STS), mid-term survival (MTS), and long-term survival (LTS). When validated on an additional cohort of 30 studies (11 STS, 9 MTS, 10 LTS), our results revealed that the most discriminative features of HES were also able to distinguish STS from LTS p=0.003).
[7]	Prognosis / OS	MGMT promoter methylation		Five radiomics features were selected to construct the radiomics signature and displayed the best performance with AUC of 0.94 and 0.86 in the primary and validation cohorts, respectively, which outweigh the performances of the clinical signature and fusion signature. With a median follow-up time of 32.4 months, the radiomics signature stratified the glioma patients into two risk groups with significantly different prognoses (p = 0.04). The radiomics signature performed the best among the three signatures in predicting the MGMT promoter methylation status.
[8]	Prognosis / OS	MGMT promoter methylation		Twenty-two radiomic features correlated with prognosis were used to successfully stratify patients into high-risk and low-risk groups (p=0.004, Log-rank test). The radiomic high- and low-risk stratification and pMGMT status were independent prognostic factors. The predictive accuracy of the pMGMT methylation status was 67% when modeled by two significant radiomic features. A significant survival difference was observed among the combined high-risk group, combined intermediate-risk group (this group consists of radiomic low risk and pMGMT- unmet or radiomic high risk and pMGMT-met), and combined low-risk group (p=0.0003, Log-rank test).
[9]	Prognosis / OS	IDH mutation		Radiomic MRI features were used to predict IDH1/IDH2 genotype in grade II-IV astrocytomas. The radiomics nomogram based on the radiomics signature and age performed better than the clinic-radiological model (training cohort, AUC = 0.913 and 0.817; validation cohort, AUC = 0.900 and 0.804). Additionally, the survival analysis showed that prognostic values of the radiomics nomogram and IDH genotype were similar (log-rank test, p < 0.001; C-index = 0.762 and 0.687; z-score test, p = 0.062).
[10]	Prognosis / OS		Lysosomal activity and autophagy (Cluster 1), chemotaxis and pro-inflammatory response (Cluster 2), activity in the MAPK pathway (Cluster 3)	GBM patients were clustered using the selected radiomics features. This resulted in 57 patients in Cluster 1 (38.8%), 67 patients in Cluster 2 (45.6%), and 23 patients in Cluster 3(15.6%). Cluster 1 was associated with heterogeneous enhancement, cluster 2 was associated with RIM-enhancing necrotic, and cluster 3 was associated with cystic features. There was a significant difference between survivals in the three clusters.
[11]	Prognosis / OS	LGG with mutant IDH1, ATRX, TERT, and MGMT promoter methylation (Cluster 1); Overexpressed PD-1, PD-L1, and	Different immune infiltration patterns between C1 and C2 with various upregulated immune cells in C1.	A total of 6 categories, including 318 radiomic features were significantly correlated with the overall survival of glioma patients. Two subgroups with distinct prognoses were separated by consensus clustering of radiomic features that were significantly associated with survival. The Kaplan–Meier plot showed that patients in cluster 2 suffered an inferior OS compared to patients in cluster 1 (p <0.0001).

		CTLA4 mRNA (Cluster 2)		
[12]	Prognosis / OS	IDH mutation		Compared to the random survival forest model (RSF from the non-imaging prognostic parameters, the addition of radiomic features significantly improved the overall survival prediction accuracy with the RSF (iAUC, 0.627 vs. 0.709; difference, 0.097; 95% CI, 0.003–0.209). Of the non-imaging prognostic factors, the IDH mutation status was the most important feature.
[13]	Prognosis / PFS	MGMT promoter methylation and IDH mutation	Cell differentiation, cell adhesion, angiogenesis	A combination of Radiomic Risk Score (RRS) with clinical (age and gender) and molecular features (MGMT and IDH status) resulted in a concordance index of 0.81 (P < 0.0001) on training and 0.84 (P 1= 0.03) on the test set. Radiogenomic analysis revealed associations of RRS features with signaling pathways for cell differentiation, cell adhesion, and angiogenesis, which contribute to chemoresistance in GBM. The survival risk score created using radiomic features from Gd-T1w MRI was found to be statistically significantly different across the "low-risk" and "high-risk" groups both on the training (P < 0.001, n = 130) and the holdout test sets (P = 0.03, n = 73). A significant association of prognostic radiomic features and molecular signaling pathways buy high BMP4 expression was associated with a better prognosis.
[14]	Prognosis / OS	Key genes not listed in the article	Immune, proliferative, treatment responsive, and cellular functions	The radiomics signature was associated with overall survival (hazard ratio [HR], 3.68; 95% CI: 2.08, 6.52; P = 0.001) in the radiomics validation subset. Four types of prognostic radiomics phenotypes were correlated with distinct pathways: immune, proliferative, treatment responsive, and cellular functions (false-discovery rate, 0.10). Thirty radiomics-correlated genes were identified. The prognostic significance of the RadGene score was confirmed in an external test set (HR, 2.02; 95% CI: 1.19, 3.41; P = 0.01) and a TCGA test set (HR, 1.43; 95% CI: 1.001, 2.04; P = 0.048). The radiomics-associated pathways and key genes can be replicated in an external test set.
[15]	Prognosis / PFS and OS	DNA copy-number subtypes (CN1, CN2, CN3)		CN2-subtype was associated with the shortest median PFS (p <0.001) and OS (p <0.001). All three RR models showed good discrimination and calibration. Decision curve analysis indicated that all RR models were clinically useful. The average accuracy of the ten-fold cross-validation was 92.8% for CN2-subtype, 72.6% for CN1-subtype, and 79.0% for CN3-subtype.

Abbreviations: ADC: Apparent diffusion coefficient, AUC: area under the curve, ATRX: X-linked helicase II, BMP4: Bone morphogenetic protein 4, CI: confidence interval, C-index: Concordance index CTLA4: cytotoxic T lymphocyte antigen 4, GBM: glioblastoma multiforme, HR: hazard ratio, IDH mutation: isocitrate dehydrogenase, MGMT methylation status: O(6)-methylguanine-DNA methyltransferase promoter methylation status, MAPK: Mitogen-activated protein kinase, MRI: Magnetic resonance imaging, OS: overall survival, PD-1: programmed cell death protein 1, PD-L1: Programmed death-ligand 1, PFS: progression-free survival, RR: radiomic and radiographic features, TCGA: The Cancer Genome Atlas program, TERT: Telomerase reverse transcriptase, TMZ: Temozolomide.

Table S2. Radiogenomics in practice - What can genetic sciences do for clinical imaging in patients with Lung and Head & Neck Cancers

[Ref.]	Utility/ Outcome Measured	Mutations/ Genes	Molecular /Biological Processes	Main Results or Finding
[16]	Prognosis / OS		cell cycling pathways	They found significant associations between the signature features and gene-expression patterns. The radiomic features were significantly associated with different biologic gene sets, demonstrating that radiomic features probe different biologic mechanisms. Both intratumoral heterogeneity features in the signature were strongly correlated with cell cycling pathways, indicating an increased proliferation for more heterogeneous tumors.
[17]	Treatment Response	EGFR mutation		On the baseline-scan, radiomic-feature Laws-Energy was significantly predictive for EGFR-mutation status (AUC = 0.67, p = 0.03), while volume (AUC = 0.59, p = 0.27) and diameter (AUC = 0.56, p = 0.46) were not. Although no features were predictive on the post-treatment scan (p > 0.08), the change in features between the two scans was strongly predictive (significant feature AUC-range = 0.74–0.91) for response to Gefitinib.
[18]	Prognosis / DFS and OS		apoptosis and proliferation genetic pathway, redox stress pathway	A DFS prediction model was made, and ROC curves estimated the AUC to be 0.866. The following features were significantly associated with DFS: N descriptor, kurtosis, surface area and spherical distortion. A prediction model of OS had AUc = 0.674 and was significantly associated with age, T descriptor, interquartile range (IQR), HU at the 25th percentile, and HU at the 97.5th percentile. Range and right lung volume were significantly associated with alternation of apoptosis and proliferation genetic pathway (p = 0.03, and p = 0.03). Energy was associated with the redox stress pathway (p = 0.06). None of the clinic radiological features showed any significant association with the alteration of differentiation and chromatin remodelers' pathway.
[19]	Prognosis / DFS		genes related to metabolic processes and the immune system	The survival analyses showed a significant difference between the radiomic high and low-risk groups (P<0.001). The pre-ranked GSEA showed the strongest associations between the radiomics signature and various genes related to metabolic processes and the immune system. The radiomics nomogram showed good discrimination performance (C-index: 0.713; 95% CI: 0.646–0.780) in the validation cohort. Patients with a low-risk score showed no survival difference with or without adjuvant chemotherapy (P=0.7), patients with high risk obtained a favorable response to adjuvant chemotherapy (P=0.04).
[20]	Prognosis / OS	ctDNA TP53 mutations		Unsupervised clustering of radiomic signatures resulted in two clusters that were associated with ctDNA TP53 mutations (p = 0.03) and changes in cfDNA concentration after 2 weeks of chemoradiation (p = 0.02). The radiomic features dissimilarity (HR = 0.56; p = 0.05), joint entropy (HR = 0.56; p = 0.04), sum entropy (HR = 0.53; p = 0.02) and normalized inverse difference (HR = 1.77; p = 0.05) were associated with overall survival.

[21]	Prognosis + Treatment Response / PFS, TTF, and DCB	EGFR mutation	An 18F-FDG-PET/CT-based deep learning model demonstrated high accuracy in EGFR mutation status prediction across patient cohorts from different institutions. A deep learning score (EGFR-DLS) was significantly and positively associated with longer PFS in patients treated with EGFR-TKIs, while EGFR-DLS is significantly and negatively associated with higher durable clinical benefit, reduced hyperprogression, and longer PFS among patients treated with immune checkpoint inhibitors.
[22]	Prognosis / PFS and OS	FOXF2, TBX4, LOC285043, and TM4SF18	They created a novel radiomics model that identified a high-risk group of early-stage patients associated with poor outcomes. FOXF2, TBX4, and TM4SF18 genes were significantly associated with the model.
[23]	Prognosis / Relapse + Histotype	CXXC4, PAK3, TP63, EPHA10, FBN2, and IL1RAP	By using machine learning-based software (RULe eXtractor 4.0) they found several radiomic-based and genomic-based rules to predict the histotype. They showed a modest ability of PET radiomic features to predict relapse, while they identified a robust gene expression signature able to predict patient relapse correctly. The best-performing model combines radiomic and genomic data with an AUC of 0.87 in predicting relapse. It was based on genes CXXC4, PAK3, and GHR gene and radiomic feature LRLGE. The best performing combined model for predicting histotype consisted of the two genes HIF1A and TP63. No correlation was found between radiomic features/genomic data and relapse, but TP63, EPHA10, FBN2, and IL1RAP is associated to histotype (adenocarcinoma / squamous cell cancer).

Abbreviations: AUC: area under the curve, CI: confidence interval, C-index: Concordance index, CT: computerized tomography, DCB: Durable clinical benefit, DFS: Disease-free survival, EGFR: epidermal growth factor receptor, FDG: fluorodeoxyglucose, GSEA: Gene Set Enrichment Analysis, HR: hazard ratio, HU: Hounsfield units, OS: overall survival, PET: positron emission tomography, PFS: progression-free survival, ROC: receiver operator characteristic curve, TTF: time-to-treatment failure

Table S3. Radiogenomics in practice - What can genetic sciences do for clinical imaging in patients with Breast, Ovarian, and Endometrial Cancer

[Ref.]	Utility/ Outcome Measured	Mutations/ Genes	Molecular /Biological Processes	Main Results or Finding
Breast Cancer				
[24]	Risk Assessment/ Risk of recurrence	Multigene assay: 70-gene MammaPrint microarray assay, 21-gene Oncotype DX assay, and the 50-gene PAM50 assay	MammaPrint and Oncotype DX were applied to the messenger RNA sequencing data	Multiple linear regression analyses demonstrated significant associations ($R2 = 0.25–0.32$, $r = 0.5–0.56$, $p < 0.001$) between radiomics signatures and multigene assay recurrence scores. Important radiomics features included tumor size and enhancement texture, which indicated tumor heterogeneity. Use of radiomics in the task of distinguishing between good and poor prognosis yielded AUC of 0.88 (SE 0.05), 0.76 (SE 0.06), 0.68 (SE 0.08), and 0.55 (SE 0.09) for MammaPrint, Oncotype DX for predicting breast cancer recurrence, PAM50 for identifying risk of relapse based on subtype, and PAM50 risk of relapse based on subtype and proliferation, respectively, with all but the latter showing statistical difference from chance.
[25]	Prognosis / PFS and OS	Genes from the Tumor-adjacent Parenchymal Gene Module and 73 Genes in the Signature for the Parenchymal Image Feature	Tumor necrosis factor (TNF) signaling pathway	The tumor necrosis factor signaling pathway was identified as the top enriched pathway (hypergeometric P, .0001) among genes associated with the image feature. A 73-gene signature based on the tumor profiles in TCGA achieved a good association with the tumor-adjacent parenchymal image feature ($R2 = 0.873$), which stratified patients into groups regarding recurrence-free survival (log-rank $P = .029$) and overall survival (log-rank $P = .042$) in an independent TCGA cohort. The prognostic value was confirmed in another independent cohort (Gene Expression Omnibus GSE 1456), with log-rank $P = .00058$ for recurrence-free survival and log-rank $P = .0026$ for overall survival.
[26]	Response to Treatment / pCR	Overexpression of the HER2 gene	HER2 is a member of ErbB family of receptor tyrosine kinases	The overall rate of pathological complete response (pCR) was 60.5% The final model to predict pCR included six MRI parameters (two clinical, four radiomic) for a sensitivity of 86.5% (32/37), specificity of 80.0% (20/25), and diagnostic accuracy of 83.9% (52/62). The final model to predict HER2 heterogeneity utilized three MRI parameters (two clinical, one radiomic) for a sensitivity of 99.3% (277/279), specificity of 81.3% (26/32), and diagnostic accuracy of 97.4% (303/311).
Ovarian Cancer				
[27]	Prognosis / OS	Amplification of CCNE1 cyclin E1 gene	CLOVAR (Classification of Ovarian Cancer transcriptomic profiles)	Of the 12 inter-site texture heterogeneity metrics evaluated, those capturing the differences in texture similarities across sites were associated with shorter overall survival (inter-site similarity entropy, similarity level cluster shade, and inter-site similarity level cluster prominence; $p \leq 0.05$) and incomplete surgical resection (similarity level cluster shade, inter-site similarity level cluster prominence and inter-site cluster variance; $p \leq 0.05$). Neither the total number of disease sites per patient nor the overall tumor volume per patient was associated with overall survival. Amplification of 19q12 involving cyclin E1 gene (CCNE1) predominantly occurred in patients with more heterogeneous inter-site textures.

[28]	Prognosis / PFS and OS	BRCA mutation		Higher inter-site cluster variance (SCV) was associated with lower PFS (p = 0.006) and OS (p = 0.003). Higher inter-site cluster prominence (SCP) was associated with lower PFS (p = 0.02), and higher inter-site cluster entropy (SE) correlated with lower OS (p = 0.01). Higher values of all three metrics were significantly associated with lower complete surgical resection status in BRCA-negative patients (SE p = 0.039m SCV p = 0.006, SCP p = 0.02), but not in BRCA-positive patients (SE p = 0.7, SCV p = 0.91, SCP p = 0.67). None of the metrics were able to distinguish between BRCA mutation carrier and non-mutation carrier.
[29]	Prognosis / PFS and OS		Extracellular-matrix (ECM)−receptor interaction and focal adhesion pathways (high RPV tumors); Proliferation and DNA damage response (DDR) pathways (low RPV tumors)	Four descriptors; "Radiomic Prognostic Vector" (RPV) reliably identifies the 5% of patients with median overall survival of less than 2 years. RPV consists of four radiomic features: (a) FD_max_25HUgl (coefficient: −0.0876), (b) GLRLM_SRLGLE_LLL_25HUgl (coefficient: 0.0869), (c) NGTDM_Contra_HLL_25HUgl (coefficient: 0.165), and (d) FOS_Imedian_LHH (coefficient: 0.250). All the features appear to have approximately even weighted and relate to tumor macro-architecture at the 25 Hounsfield Unit gray level (and discrete wavelet filters). In biological terms, the individual components of RPV combine to define the tumor mesoscopic structure: (a) maximal fractal dimension of the tumor and its microenvironment, which was negatively correlated with survival, together with the following positively correlated features; (b) proportions of runs that have short lengths in the low pass filtered image; a function which gives coarse low-density textures, e.g. intermixed fibrotic stroma and tumor cells; (c) the average visual contrast across the tumor weighted by sharpening in the x-axis and blurring in the y and z axes reflecting local heterogeneity, and (d) the median of the distribution of voxel intensities across the entire tumor weighted by blurring in the x-axis and sharpening in the y and z axes, reflecting global heterogeneity, respectively.
Endometrium Cancer				
[30]	Prognosis / RFS and DSS	Copy-number high or p53-altered	Upregulated: HSPA5, GATA3, and HSP90AA1; Downregulated: SCGB2A1, GSTK1, MMP7, GDF15, ANXA1, SAT1, CNDP2, and PBX1	Radiomic features identified two distinct patient clusters: cluster 1 and cluster 2. Cluster 2 had subclusters 2a and 2b. Patients in cluster 2 had significantly reduced disease-specific survival (p < 0.001). Furthermore, patients in clusters 1, 2a, and 2b had significantly different disease-specific and recurrence-free survival (overall p < 0.001). A gene signature related to the most aggressive clusters including an 11-gene high-risk signature was defined and associated with poor survival. A subsequent dataset using machine learning-based tumor segmentation, instead of manual segmentation, also showed that patients in cluster 2 had significantly reduced disease-specific (p < 0.0001) and recurrence-free survival (p=0.003). In the ML-cohort low-risk gene signature tended to be more frequent within cluster 1 than 2 (p = 0.089).

Abbreviations: AUC: area under the curve, BRCA: breast cancer gene, DSS: Disease-specific survival, GSE: Gene Expression Omnibus, HER2: human epidermal growth factor receptor 2, MFS: Metastasis-free survival, MRI: Magnetic resonance imaging, OS: overall survival, pCR: pathologic complete response, PFS: progression-free survival, RFS: Recurrence-free survival, TCGA: The Cancer Genome Atlas program.

Table S4. Radiogenomics in practice - What can genetic sciences do for clinical imaging in patients with Urogenital Cancers

[Ref.]	Utility/ Outcome Measured	Mutations/ Genes	Molecular /Biological Processes	Main Results or Finding
Renal Cell Carcinoma				
[31]	Prognosis / MFS and OS	Non-specific gene names are listed in the text	Eukaryotic translation elongation, initiation, and termination; ECM–receptor interaction, Regulation of actin cytoskeleton; Focal adhesion, PI3K/AKT, and integrin signaling pathway	Three radiomic features (INNER_MaxProb_GLCM, OUTER_Energy_Hist and Under80HURatio) showed positive weights (0.0101, OR: 1.0101; 0.7281, OR: 2.0711; 0.5538, OR: 1.7399), and one feature (INNER_Min-hist) showed negative weights (-0.1947, OR: 0.8231) towards postsurgical metastasis. In the discovery cohort, the radiomic risk score of MFS had an optimal cutoff of 0.4176 (HR=8.2954, 95%CI = 2.0957-32.8362, p = 0.0077), and in the validation cohort optimal cutoff of 1.3128 (HR=2.2264 x 10^5, 95 %CI = 1.3878 x 10^3 - 3.5719 x 10^7, p = 0.0005). Trait-associated genes was found correlated to four features.
[32]	Prognosis / OS	Hypoxia-related genes (IFT57, PABPN1, RNF10, RNF19B, UBE2T		The radiogenomics biomarker consisting of 13 radiomic features that were optimal predictors of hypoxia-gene signature expression level (low- or high-risk) demonstrated AUC values of 0.91 in both the training and validation groups respectively. IFT57 and RNF19B were identified as low-risk prognostic genes whereas PABPN1, RNF10, and UBE2T were considered high-risk prognostic factors. In the independent validation cohort, the radiogenomics biomarker was significantly associated with prognosis in patients with ccRCC (p = 0.0059).
[33]	Prognosis / PFI		Genes were most strongly related to immunological synapse and chemokine activity	For the radiomic signature, the AUC for the training cohort was 0.897 and for the validation cohort 0.712. The C-index value of the radiomic signature was 0.861 (95 % CI 0.789-0.927). The Kaplan-Meier survival curve analysis of the radiomic signature revealed that the PFI of the high-risk group was dramatically shorter than that of the low-risk group (P < 0.05). The functional analysis indicated that radiomic signature was significantly associated with T cell activation.
[34]	Prognosis / PFI and OS	VHL, MUC16, FBN2, FLG		Radiomic profiling revealed three ccRCC subtypes (C1, C2, C3) with distinct clinic-pathological features and prognoses. VHL, MUC16, FBN2, and FLG were found to have different mutation frequencies in these radiomic subtypes. While VHL had a high mutation rate in all subtypes, it was less frequently observed in the C1 subtype than in the C2 and C3 subtypes. In addition, FLG, MUC16, and FBN2 mutations were specifically observed in the C1, C2, and C3 subtypes, respectively. The C1 subtype had significantly lower OS (p = 0.027) and PFI (p = 0.002) than the C2 and C3 subtypes.
[35]	Prognosis / OS	VHL mutation	Epithelial-mesenchymal transition (EMT) score, purity, mRNA subtype, miRNA subtype	A strong statistical correlation (R^2 = 0.83) between the feature crosses and genomics characteristics was shown. The SEM confirmed significant association between CT feature, pathological (β = -0.75), and molecular subtype (β = -0.30). Patients with high CT texture feature such as the first-order statistics t_first-order_TE (total energy of tumor), r_glszm_LALGLE values tended to have a poor prognosis.

[36]	Prognosis / OS	VHL, BAP1, PBRM1, SETD2	Molecular subtypes reflected by mRNA patterns	Using radiomic features, random forest algorithm showed good capacity to identify the mutations VHL (AUC=0.971), BAP1 (AUC=0.955), PBRM1 (AUC=0.972), SETD2 (AUC=0.949). In the validation set, the AUC of radiomics model was significantly higher than the genomics model at 5-year (0.775 vs. 0.684, p=0.030) time point. The integrative model of radiomics and genomics achieved better predictive performance, the 1-year, 3-year, and 5-year AUCs of which were 0.807, 0.814, and 0.784. The increase in AUC was compared with radiomics and genomics models (all p < 0.05). The high-risk groups in the radiomics model (HR = 3.62, 95%CI: 2.04-6.73, p = 0.002) and radiomics-genomics model (HR = 3.93, 95%CI: 2.27-12.12, p = 0.017) were significantly related with poorer survival.
Bladder Cancer				
[37]	Prognosis / PFI	Non-specific gene names are listed in the text	Angiogenesis	The radiomics and transcriptomics signatures significantly stratified BLCA patients into high- and low-risk groups in terms of the progression-free interval (PFI). The two risk models remained independent prognostic factors in multivariate analyses after adjusting for clinical parameters. A nomogram was developed and showed an excellent predictive ability for the PFI in BLCA patients. Functional enrichment analysis suggested that the radiomics signature we developed could reflect the angiogenesis status of BLCA patients.
Prostate Cancer				
[38]	Risk Assessment / Risk of metastasis	Non-specific gene names are listed in the text		A total of 14 radiomics features significantly correlated with the Gleason score (highest correlation r [0.39, p [0.001). A total of 31 texture and histogram features significantly correlated with 19 gene signatures, particularly with the PORTOS (Post-Operative Radiation Therapy Outcomes Score) signature (strongest correlation r [e0.481, p [0.002). A total of 40 diffusion-weighted imaging features correlated significantly with 132 gene expression levels. Machine learning prediction models showed fair performance to predict a Gleason score of 8 or greater (AUC 0.72) and excellent performance to predict a Decipher score of 0.6 or greater (AUC 0.84).

Abbreviations: AUC: area under the curve, ccRCC: clear cell renal cell carcinoma, CI: confidence interval, CT: computerized tomography, HR: hazard ratio, MFS: Metastasis-free survival, OR: odds ratio, OS: overall survival, PFI: progression-free interval, VHL: Von Hippel-Lindau.

Table S5. Radiogenomics in practice - What can genetic sciences do for clinical imaging in patients with Gastrointestinal Tumors

[Ref.]	Utility/ Outcome Measured	Mutations/ Genes	Molecular /Biological Processes	Main Results or Finding
Esophagus Cancer				
[39]	Treatment Response / pCR		type I interferon, lymphocyte apoptosis, and natural killer cell activation pathways	The optimal intratumoral and peritumoral radiomics models yielded similar areas under the receiver operating characteristic curve of 0.730 (95% CI, 0.609-0.850) and 0.734 (0.613-0.854), respectively. The combined model was composed of 7 intratumoral and 6 peritumoral features and achieved better discriminative performance, with an AUC of 0.852 (95% CI, 0.753-0.951), an accuracy of 84.3%, sensitivity of 90.3%, and specificity of 79.5% in the test set. Gene sets associated with the combined model mainly involved lymphocyte-mediated immunity. The association of the peritumoral area with response identification might be partially attributed to type I interferon–related biological processes.
[40]	Prognosis + Treatment Response / DFS, OS and pCR	KLK8, STOX1, SPRY2, GPRC5A, IGSF10, COBL, TXNIP, EPS8, GPX3, KLK6, M1AP, ZNF483, KIF26B, SGCB, FGF4, PRCP	Non-specific pathways are listed in the text	Radiomic features that were correlated to differentially expressed (DE) genes were selected for the nomogram. The nomogram significantly stratified patients into high- and low-risk groups for disease-free survival (DFS) ($p < 0.001$). AUCs for predicting 5-year DFS were 0.912 in the training set, 0.852 in the internal test set, and 0.769 in the external test set.
Gastric Cancer				
[41]	Prognosis + Treatment Response / PFS and OS		Cell cycling pathways, chemokine signaling, and chemotherapeutic drug metabolism; drug metabolism cytochrome P450 and other enzymes	The radiomics signature had discriminative power of PFS and OS (AUCs = 0.753). The low radiomics score subgroup, obtained a terrible response to adjuvant chemotherapy (ACT), while the high score subgroup showed no significant survival difference with or without ACT. The clinical-radiomics nomogram showed association with OS at 1-, 3-, and 5- year survival and had AUCs of 0.80, 0.816, and 0.965, respectively in the prediction performance analysis. The pre-ranked GSEA showed that the significantly enriched pathways (FDR <0.1) among the top associations with these four radiomic factors were mostly correlated with drug metabolism and chemokine regulation.
Colorectal Cancer				
[42]	Prognosis / OS	BRAF mutation	BRAF tumors showed lower levels of hypoxia and more angiogenesis than wild-type BRAF tumor	Tumor radiomics texture features, including the standard deviations and the mean value of positive pixels were significantly lower in BRAF mutant tumors than in wild-type BRAF tumors at spatial scaling factors (SSFs) of 0, 4, and 6 (P = .006, P = .007, and P = .005, respectively). Patients with skewness less than or equal to 20.75 at an SSF of 0 and a mean of greater than or equal to 17.76 at an SSF of 2 showed better 5-year OS (HR, 0.53 [95%CI: 0.29, 0.94]; HR, 0.40 [95%CI: 0.22, 0.71]; log-rank P = .025 and P = .002, respectively). Tumor location (right colon vs left colon vs rectum) had no significant impact on the clinical outcome (log-rank P = .53).

Hepatocellular Carcinoma				
[43]	Risk Assessment / Early recurrence		PD-L1 protein, PD1, CTLA4 mRNA expression level	Radiomics features correlated with the expression of immunotherapy targets PD-L1 at the protein level as well as PD1 and CTLA4 at the mRNA expression level (r = -0.48-0.47, p < 0.037). Radiomics features, including tumor size, showed significant diagnostic performance for assessment of early hepatocellular carcinoma recurrence (AUC 0.76-0.80, p < 0.043), while immune-profiling and genomic features did not.

Abbreviations: AUC: area under the curve, BRAF mutation: serine-threonine kinase protein, CI: confidence interval, CTLA4: cytotoxic T lymphocyte antigen 4, DFS: Disease-free survival, FDR: false diagnostic rate, OS: overall survival pCR: pathologic complete response, PD-1: programmed cell death protein 1, PD-L1: Programmed death-ligand 1, PFS: progression-free survival, ROC: receiver operating characteristic curve.

Table S6. Radiogenomics in practice - What can genetic sciences do for clinical imaging in patients with Melanoma and Solid Tumors

[Ref.]	Utility/ Outcome Measured	Mutations/ Genes	Molecular /Biological Processes	Main Results or Finding
Melanoma				
[44]	Prognosis / OS	BRAF mutation	CD8 Expression	Two radiomics markers are associated with poor outcomes and immune disturbances in melanoma patients. Analysis indicated patients with high standard deviation or high mean of positive pixels (MPP) had worse progression-free survival (P = 0.00047 and P = 0.0014, respectively) and worse overall survival (P = 0.0223 and P = 0.0465, respectively). Whole-exome sequencing showed high MPP was associated with BRAF mutation status (P = 0.0389). RNA sequencing indicated patients with immune "cold" signatures had worse survival, which was associated with the CT biomarker, MPP4 (P = 0.0284). Multiplex immunofluorescence confirmed a correlation between CD8 expression and image biomarkers (P = 0.0028).
Solid Tumors*				
[45]	Prognosis + Treatment Response / PFI, OS and Response to Treatment		Gene expression signature of CD8 cells	The developed radiomic signature for CD8 cells was validated with the gene expression signature of CD8 cells (AUC=0.67, 95% CI 0.57-0.77; p=0.0019.) In patients treated with anti-PD-1 and PD-L1, a high baseline radiomic score was associated with a higher proportion of patients who achieved an objective response at 3 months (p=0.049) and a higher proportion of patients who had an objective response (p=0.025) or stable disease (p=0.013) at 6 months. A high baseline radiomic score was associated with improved overall survival in univariate (HR 0.58, 95% CI 0.39-0.87; p=0.0081) and multivariate analyses (HR 0.52, 95% CI 0.35-0.79; p=0.0022)

Abbreviations: AUC: area under the curve, BRAF mutation: serine-threonine kinase protein, CI: confidence interval, HR: hazard ratio, OS: overall survival PD-1: programmed cell death protein 1, PD-L1: Programmed death-ligand 1, PFI: progression-free interval.

*Head and neck-squamous-cell carcinoma (HNSC), lung squamous cell carcinoma (LUSC), lung adenocarcinoma (LUAD), liver hepatocellular carcinoma (LIHC), bladder endothelial carcinoma (BLCA), sarcomas, etc.

References

1. Grossmann, P.; Gutman, D. A.; Dunn, W. D., Jr.; Holder, C. A.; Aerts, H. J., Imaging-genomics reveals driving pathways of MRI derived volumetric tumor phenotype features in Glioblastoma. *BMC Cancer* 16, 611.
2. Dextraze, K.; Saha, A.; Kim, D.; Lehrer, M.; Narang, S.; Rao, D.; Ahmed, S.; Madhugiri, V.; Fuller, C. D.; Kim, M. M.; Krishnan, S.; Rao, G.; Rao, A., Spatial habitats from multiparametric MR imaging are associated with signaling pathway activities and survival in glioblastoma. *Oncotarget* **2017,** 8, 112992-113001.
3. Liao, X.; Cai, B.; Tian, B.; Luo, Y.; Song, W.; Li, Y., Machine-learning based radiogenomics analysis of MRI features and metagenes in glioblastoma multiforme patients with different survival time. *Journal of Cellular and Molecular Medicine* 23, 4375-4385.
4. Wei, J.; Yang, G.; Hao, X.; Gu, D.; Tan, Y.; Wang, X.; Dong, D.; Zhang, S.; Wang, L.; Zhang, H.; Tian, J., A multi-sequence and habitat-based MRI radiomics signature for preoperative prediction of MGMT promoter methylation in astrocytomas with prognostic implication. *Eur Radiol* 29, (2), 877-888.
5. Qian, Z.; Li, Y.; Sun, Z.; Fan, X.; Xu, K.; Wang, K.; Li, S.; Zhang, Z.; Jiang, T.; Liu, X.; Wang, Y., Radiogenomics of lower-grade gliomas: a radiomic signature as a biological surrogate for survival prediction. *Aging (Albany NY)* 10, (10), 2884-2899.
6. Beig, N.; Patel, J.; Prasanna, P.; Hill, V.; Gupta, A.; Correa, R.; Bera, K.; Singh, S.; Partovi, S.; Varadan, V.; Ahluwalia, M.; Madabhushi, A.; Tiwari, P., Radiogenomic analysis of hypoxia pathway is predictive of overall survival in Glioblastoma. *Sci Rep* 8, (1), 7.
7. Kong, Z.; Lin, Y.; Jiang, C.; Li, L.; Liu, Z.; Wang, Y.; Dai, C.; Liu, D.; Qin, X.; Wang, Y.; Liu, Z.; Cheng, X.; Tian, J.; Ma, W., (18)F-FDG-PET-based Radiomics signature predicts MGMT promoter methylation status in primary diffuse glioma. *Cancer Imaging* **2019,** 19, (1), 58.
8. Sasaki, T.; Kinoshita, M.; Fujita, K.; Fukai, J.; Hayashi, N.; Uematsu, Y.; Okita, Y.; Nonaka, M.; Moriuchi, S.; Uda, T.; Tsuyuguchi, N.; Arita, H.; Mori, K.; Ishibashi, K.; Takano, K.; Nishida, N.; Shofuda, T.; Yoshioka, E.; Kanematsu, D.; Kodama, Y.; Mano, M.; Nakao, N.; Kanemura, Y., Radiomics and MGMT promoter methylation for prognostication of newly diagnosed glioblastoma. *Sci Rep* 9, (1), 14435.
9. Tan, Y.; Mu, W.; Wang, X. C.; Yang, G. Q.; Gillies, R. J.; Zhang, H., Improving survival prediction of high-grade glioma via machine learning techniques based on MRI radiomic, genetic and clinical risk factors. *Eur J Radiol* **2019,** 120, 108609.
10. Choi, S. W.; Cho, H. H.; Koo, H.; Cho, K. R.; Nenning, K. H.; Langs, G.; Furtner, J.; Baumann, B.; Woehrer, A.; Cho, H. J.; Sa, J. K.; Kong, D. S.; Seol, H. J.; Lee, J. I.; Nam, D. H.; Park, H., Multi-habitat radiomics unravels distinct phenotypic subtypes of glioblastoma with clinical and genomic significance. *Cancers* **2020,** 12, 1-15.
11. Lin, P.; Peng, Y. T.; Gao, R. Z.; Wei, Y.; Li, X. J.; Huang, S. N.; Fang, Y. Y.; Wei, Z. X.; Huang, Z. G.; Yang, H.; Chen, G., Radiomic profiles in diffuse glioma reveal distinct subtypes with prognostic value. *J Cancer Res Clin Oncol* **2020,** 146, (5), 1253-1262.
12. Choi, Y. S.; Ahn, S. S.; Chang, J. H.; Kang, S. G.; Kim, E. H.; Kim, S. H.; Jain, R.; Lee, S. K., Machine learning and radiomic phenotyping of lower grade gliomas: improving survival prediction. *Eur Radiol* **2020,** 30, (7), 3834-3842.
13. Beig, N.; Bera, K.; Prasanna, P.; Antunes, J.; Correa, R.; Singh, S.; Bamashmos, A. S.; Ismail, M.; Braman, N.; Verma, R.; Hill, V. B.; Statsevych, V.; Ahluwalia, M. S.; Varadan, V.; Madabhushi, A.; Tiwari, P., Radiogenomic-based survival risk stratification of tumor habitat on Gd-T1w MRI is associated with biological processes in glioblastoma. *Clinical Cancer Research* **2020,** 26, 1866-1876.
14. Sun, Q.; Chen, Y.; Liang, C.; Zhao, Y.; Lv, X.; Zou, Y.; Yan, K.; Zheng, H.; Liang, D.; Li, Z. C., Biologic Pathways Underlying Prognostic Radiomics Phenotypes from Paired MRI and RNA Sequencing in Glioblastoma. *Radiology* **2021,** 301, (3), 654-663.

15. Wan, Y.; Zhou, S.; zhang, Y.; Deng, X.; Xu, L., Radiomic Analysis of Contrast-Enhanced MRI Predicts DNA Copy-Number Subtype and Outcome in Lower-Grade Gliomas. *Academic Radiology.* **2021**.
16. Aerts, H. J.; Velazquez, E. R.; Leijenaar, R. T.; Parmar, C.; Grossmann, P.; Carvalho, S.; Bussink, J.; Monshouwer, R.; Haibe-Kains, B.; Rietveld, D.; Hoebers, F.; Rietbergen, M. M.; Leemans, C. R.; Dekker, A.; Quackenbush, J.; Gillies, R. J.; Lambin, P., Decoding tumour phenotype by noninvasive imaging using a quantitative radiomics approach. *Nat Commun* **2014,** 5, 4006.
17. Aerts, H. J.; Grossmann, P.; Tan, Y.; Oxnard, G. R.; Rizvi, N.; Schwartz, L. H.; Zhao, B., Defining a Radiomic Response Phenotype: A Pilot Study using targeted therapy in NSCLC. *Sci Rep* **2016,** 6, 33860.
18. Bak, S. H.; Park, H.; Lee, H. Y.; Kim, Y.; Kim, H. L.; Jung, S. H.; Kim, H.; Kim, J.; Park, K., Imaging genotyping of functional signaling pathways in lung squamous cell carcinoma using a radiomics approach. *Sci Rep* **2018,** 8, (1), 3284.
19. Xie, D.; Wang, T. T.; Huang, S. J.; Deng, J. J.; Ren, Y. J.; Yang, Y.; Wu, J. Q.; Zhang, L.; Fei, K.; Sun, X. W.; She, Y. L.; Chen, C., Radiomics nomogram for prediction disease-free survival and adjuvant chemotherapy benefits in patients with resected stage I lung adenocarcinoma. *Transl Lung Cancer Res* **2020,** 9, (4), 1112-1123.
20. Lafata, K. J.; Corradetti, M. N.; Gao, J.; Jacobs, C. D.; Weng, J.; Chang, Y.; Wang, C.; Hatch, A.; Xanthopoulos, E.; Jones, G.; Kelsey, C. R.; Yin, F. F., Radiogenomic Analysis of Locally Advanced Lung Cancer Based on CT Imaging and Intratreatment Changes in Cell-Free DNA. *Radiol Imaging Cancer* **2021,** 3, (4), e200157.
21. Mu, W.; Jiang, L.; Zhang, J.; Shi, Y.; Gray, J. E.; Tunali, I.; Gao, C.; Sun, Y.; Tian, J.; Zhao, X.; Sun, X.; Gillies, R. J.; Schabath, M. B., Non-invasive decision support for NSCLC treatment using PET/CT radiomics. *Nat Commun* **2020,** 11, (1), 5228.
22. Perez-Morales, J.; Tunali, I.; Stringfield, O.; Eschrich, S. A.; Balagurunathan, Y.; Gillies, R. J.; Schabath, M. B., Peritumoral and intratumoral radiomic features predict survival outcomes among patients diagnosed in lung cancer screening. *Scientific reports* **2020,** 10, 10528.
23. Kirienko, M.; Sollini, M.; Corbetta, M.; Voulaz, E.; Gozzi, N.; Interlenghi, M.; Gallivanone, F.; Castiglioni, I.; Asselta, R.; Duga, S.; Soldà, G.; Chiti, A., Radiomics and gene expression profile to characterise the disease and predict outcome in patients with lung cancer. *Eur J Nucl Med Mol Imaging* **2021,** 48, (11), 3643-3655.
24. Li, H.; Zhu, Y.; Burnside, E. S.; Drukker, K.; Hoadley, K. A.; Fan, C.; Conzen, S. D.; Whitman, G. J.; Sutton, E. J.; Net, J. M.; Ganott, M.; Huang, E.; Morris, E. A.; Perou, C. M.; Ji, Y.; Giger, M. L., MR Imaging Radiomics Signatures for Predicting the Risk of Breast Cancer Recurrence as Given by Research Versions of MammaPrint, Oncotype DX, and PAM50 Gene Assays. *Radiology* **2016,** 281, 382-391.
25. Wu, J.; Li, B.; Sun, X.; Cao, G.; Rubin, D. L.; Napel, S.; Ikeda, D. M.; Kurian, A. W.; Li, R., Heterogeneous Enhancement Patterns of Tumor-adjacent Parenchyma at MR Imaging Are Associated with Dysregulated Signaling Pathways and Poor Survival in Breast Cancer. *Radiology* **2017,** 285, (2), 401-413.
26. Bitencourt, A. G. V.; Gibbs, P.; Rossi Saccarelli, C.; Daimiel, I.; Lo Gullo, R.; Fox, M. J.; Thakur, S.; Pinker, K.; Morris, E. A.; Morrow, M.; Jochelson, M. S., MRI-based machine learning radiomics can predict HER2 expression level and pathologic response after neoadjuvant therapy in HER2 overexpressing breast cancer. *EBioMedicine* **2020,** 61, 103042.
27. Vargas, H. A.; Veeraraghavan, H.; Micco, M.; Nougaret, S.; Lakhman, Y.; Meier, A. A.; Sosa, R.; Soslow, R. A.; Levine, D. A.; Weigelt, B.; Aghajanian, C.; Hricak, H.; Deasy, J.; Snyder, A.; Sala, E., A novel representation of inter-site tumour heterogeneity from pre-treatment computed tomography textures classifies ovarian cancers by clinical outcome. *Eur Radiol* **2017,** 27, (9), 3991-4001.

28. Meier, A.; Veeraraghavan, H.; Nougaret, S.; Lakhman, Y.; Sosa, R.; Soslow, R. A.; Sutton, E. J.; Hricak, H.; Sala, E.; Vargas, H. A., Association between CT-texture-derived tumor heterogeneity, outcomes, and BRCA mutation status in patients with high-grade serous ovarian cancer. *Abdom Radiol (NY)* **2018,** 44, (6), 2040-2047.
29. Lu, H.; Arshad, M.; Thornton, A.; Avesani, G.; Cunnea, P.; Curry, E.; Kanavati, F.; Liang, J.; Nixon, K.; Williams, S. T.; Hassan, M. A.; Bowtell, D. D. L.; Gabra, H.; Fotopoulou, C.; Rockall, A.; Aboagye, E. O., A mathematical-descriptor of tumor-mesoscopic-structure from computed-tomography images annotates prognostic- and molecular-phenotypes of epithelial ovarian cancer. *Nat Commun* **2019,** 10, (1), 764.
30. Hoivik, E. A.; Hodnel; , E.; Dybvik, J. A.; Wagner-Larsen, K. S.; Fasmer, K. E.; Berg, H. F.; Halle, M. K.; Haldorsen, I. S.; Krakstad, C., A radiogenomics application for prognostic profiling of endometrial cancer. *Commun Biol* **2021,** 4, (1), 1363.
31. Lee, H. W.; Cho, H. H.; Joung, J. G.; Jeon, H. G.; Jeong, B. C.; Jeon, S. S.; Lee, H. M.; Nam, D. H.; Park, W. Y.; Kim, C. K.; Seo, S. I.; Park, H., Integrative radiogenomics approach for risk assessment of post-operative metastasis in pathological T1 renal cell carcinoma: A pilot retrospective cohort study. *Cancers* **2020,** 12.
32. Gao, J.; Ye, F.; Han, F.; Wang, X.; Jiang, H.; Zhang, J., A Novel Radiogenomics Biomarker Based on Hypoxic-Gene Subset: Accurate Survival and Prognostic Prediction of Renal Clear Cell Carcinoma. *Frontiers in Oncology* **2021,** 11.
33. Gao, Y.; Mao, Y.; Lu, S.; Tan, L.; Li, G.; Chen, J.; Huang, D.; Zhang, X.; Qiu, Y.; Liu, Y., Magnetic resonance imaging-based radiogenomics analysis for predicting prognosis and gene expression profile in advanced nasopharyngeal carcinoma. *Head Neck* **2021,** 43, (12), 3730-3742.
34. Lin, P.; Lin, Y. Q.; Gao, R. Z.; Wen, R.; Qin, H.; He, Y.; Yang, H., Radiomic profiling of clear cell renal cell carcinoma reveals subtypes with distinct prognoses and molecular pathways. *Translational Oncology* **2021,** 14.
35. Wu, K.; Wu, P.; Yang, K.; Li, Z.; Kong, S.; Yu, L.; Zhang, E.; Liu, H.; Guo, Q.; Wu, S., A comprehensive texture feature analysis framework of renal cell carcinoma: pathological, prognostic, and genomic evaluation based on CT images. *European Radiology.* **2021.**
36. Zeng, H.; Chen, L.; Wang, M.; Luo, Y.; Huang, Y.; Ma, X., Integrative radiogenomics analysis for predicting molecular features and survival in clear cell renal cell carcinoma. *Aging (Albany NY)* **2021,** 13, (7), 9960-9975.
37. Lin, P.; Wen, D. Y.; Chen, L.; Li, X.; Li, S. H.; Yan, H. B.; He, R. Q.; Chen, G.; He, Y.; Yang, H., A radiogenomics signature for predicting the clinical outcome of bladder urothelial carcinoma. *Eur Radiol* **2019,** 30, (1), 547-557.
38. Hectors, S. J.; Cherny, M.; Yadav, K. K.; Beksaç, A. T.; Thulasidass, H.; Lewis, S.; Davicioni, E.; Wang, P.; Tewari, A. K.; Taouli, B., Radiomics Features Measured with Multiparametric Magnetic Resonance Imaging Predict Prostate Cancer Aggressiveness. *J Urol* **2019,** 202, (3), 498-505.
39. Hu, Y.; Xie, C.; Vardhanabhuti, V.; Fu, J., Application of machine learning to predict neoadjuvant chemoradiotherapy treatment response in esophageal squamous cell carcinoma using computed tomography. *Diseases of the Esophagus* **2020,** 33, 3.
40. Xie, C. Y.; Hu, Y. H.; Ho, J. W. K.; Han, L. J.; Yang, H.; Wen, J.; Lam, K. O.; Wong, I. Y. H.; Law, S. Y. K.; Chiu, K. W. H.; Fu, J. H.; Vardhanabhuti, V., Using genomics feature selection method in radiomics pipeline improves prognostication performance in locally advanced esophageal squamous cell carcinoma-a pilot study. *Cancers* **2021,** 13.
41. Jin, Y.; Xu, Y.; Li, Y.; Chen, R.; Cai, W., Integrative Radiogenomics Approach for Risk Assessment of Postoperative and Adjuvant Chemotherapy Benefits for Gastric Cancer Patients. *Front Oncol* **2021,** 11, 755271.
42. Negreros-Osuna, A. A.; Parakh, A.; Corcoran, R. B.; Pourvaziri, A.; Kambadakone, A.; Ryan, D. P.; Sahani, D. V., Radiomics Texture Features in Advanced Colorectal Cancer: Correlation with BRAF Mutation and 5-year Overall Survival. *Radiol Imaging Cancer* **2020,** 2, (5), e190084.

43. Hectors, S. J.; Lewis, S.; Besa, C.; King, M. J.; Said, D.; Putra, J.; Ward, S.; Higashi, T.; Thung, S.; Yao, S.; Laface, I.; Schwartz, M.; Gnjatic, S.; Merad, M.; Hoshida, Y.; Taouli, B., MRI radiomics features predict immuno-oncological characteristics of hepatocellular carcinoma. *European Radiology* **2020,** 30, 3759-3769.
44. Aoude, L. G.; Wong, B. Z. Y.; Bonazzi, V. F.; Brosda, S.; Walters, S. B.; Koufariotis, L. T.; Naeini, M. M.; Pearson, J. V.; Oey, H.; Patel, K.; Bradford, J. J.; Bloxham, C. J.; Atkinson, V.; Law, P.; Strutton, G.; Bayley, G.; Yang, S.; Smithers, B. M.; Waddell, N.; Miles, K.; Barbour, A. P., Radiomics Biomarkers Correlate with CD8 Expression and Predict Immune Signatures in Melanoma Patients. *Mol Cancer Res* **2021,** 19, (6), 950-956.
45. Sun, R.; Limkin, E. J.; Vakalopoulou, M.; Dercle, L.; Champiat, S.; Han, S. R.; Verlingue, L.; Br; ao, D.; Lancia, A.; Ammari, S.; Hollebecque, A.; Scoazec, J. Y.; Marabelle, A.; Massard, C.; Soria, J. C.; Robert, C.; Paragios, N.; Deutsch, E.; Ferté, C., A radiomics approach to assess tumour-infiltrating CD8 cells and response to anti-PD-1 or anti-PD-L1 immunotherapy: an imaging biomarker, retrospective multicohort study. *Lancet Oncol* **2018,** 19, (9), 1180-1191.
